# Symbiotic biofilms formed by *Clostridioides difficile* and *bacteroides thetaiotaomicron* in the presence of vancomycin

**DOI:** 10.1080/19490976.2024.2390133

**Published:** 2024-08-12

**Authors:** Jingpeng Yang, Wen Rui, Saiwei Zhong, Xiaoqian Li, Wenzheng Liu, Lingtong Meng, Yanan Li, He Huang

**Affiliations:** School of Food Science and Pharmaceutical Engineering, Nanjing Normal University, Qixia District, Nanjing, China

**Keywords:** Vancomycin, *clostridioides difficile*, symbiotic biofilms, *B. thetaiotaomicron*, toxin protein, virulence genes

## Abstract

Vancomycin (VAN) treatment in *Clostridioides difficile* infection (CDI) suffers from a relatively high rate of recurrence, with a variety of reasons behind this, including biofilm-induced recurrent infections. *C. difficile* can form monophyletic or symbiotic biofilms with other microbes in the gut, and these biofilms protect *C. difficile* from being killed by antibiotics. In this study, we analyzed the ecological relationship between *Bacteroides thetaiotaomicron* and *C. difficile* and their formation of symbiotic biofilm in the VAN environment. The production of symbiotic biofilm formed by *C. difficile* and *B. thetaiotaomicron* was higher than that of *C. difficile* and *B. thetaiotaomicron* alone in the VAN environment. In symbiotic biofilms, *C. difficile* was characterized by increased production of the toxin protein TcdA and TcdB, up-regulation of the expression levels of the virulence genes *tcdA* and *tcdB*, enhanced bacterial cell swimming motility and c-di-GMP content, and increased adhesion to Caco-2 cells. The scanning electron microscope (SEM) combined with confocal laser scanning microscopy (CLSM) results indicated that the symbiotic biofilm was elevated in thickness, dense, and had an increased amount of mixed bacteria, while the fluorescence in situ hybridization (FISH) probe and plate colony counting results further indicated that the symbiotic biofilm had a significant increase in the amount of *C. difficile* cells, and was able to better tolerate the killing of the simulated intestinal fluid. Taken together, *C. difficile* and *B. thetaiotaomicron* become collaborative in the VAN environment, and targeted deletion or attenuation of host gut *B. thetaiotaomicron* content may improve the actual efficacy of VAN in CDI treatment.

## 1. Introduction

*Clostridioides difficile* is a Gram-positive anaerobic bacterium that produces spores.^1^ As a common conditional pathogen in the gut, *C. difficile* produces two pathogenic toxin proteins, A and B, which can synergistically destroy intestinal epithelial cells and induce inflammation and tissue damage under conditions of intestinal homeostatic imbalance, a phenomenon known as *C. difficile* infection (CDI).^2^ Primary *C. difficile* infection (pCDI) is usually treated with antibiotics, including the use of vancomycin (VAN), but is accompanied by a high rate of recurrence and recurrent *C. difficile* infection (rCDI) is more difficult to cure.^3^ The main reasons behind this phenomenon include the formation of recalcitrant *C. difficile* spores, the development of multi-drug resistant strains, and opportunistic microbial infections due to disturbances in gut ecology.^3^ The presence of biofilms is also one of the major causes of pCDI and rCDI, including the formation of monophyletic biofilms by *C. difficile* and the formation of symbiotic biofilms by *C. difficile* with other members in the gut.^4^ Several studies have shown that the presence of biofilm is effective in increasing the resistance of *C. difficile* to antibiotics and host immune killing.^5,6^

Recent studies have revealed that *C. difficile* can synergize with specific microbes in the gut to enhance its intestinal colonization ability and environmental resistance.^7^ For example, *Fusobacterium nucleatum* was able to aggregate with *C. difficile*, promoting its biofilm production and synergizing its colonization of the mucus layer.^7^ Clinical data suggested that CDI are often accompanied by an increase in the abundance of other potentially pathogenic enteric organisms, including *Enterococcus*, *Escherichia/Shigella*, *Salmonella*, *Staphylococcus*, *Klebsiella*, *Fusobacterium* and was accompanied by a decline in the abundance of beneficial microbes such as *Blautia* and *Bifidobacterium*.^7,8^ Notably, members of the genus *Bacteroides* also exhibit differential abundance, showing a trend of positive correlation with the CDI.^9,10^ Studies have shown that *Bacteroides* are a group of gram-negative, rod-shaped, bile-resistant, non-spore-forming bacteria that can make up as much as one-fourth of the host’s overall microbiota and that the main representative members of this genus are *Bacteroides thetaiotaomicron*, *Bacteroides vulgatus*, *Bacteroides distasonis*, *Bacteroides fragilis*, *Bacteroides ovatus*, *Bacteroides eggerthii*, *Bacteroides uniformis* and others.^9^

In our previous study, we found a highly positive correlation between *C. difficile* and *Bacteroides* abundance, especially the percentage of *B. thetaiotaomicron*, which remained high level in the gut of pCDI mice before and after VAN treatment, and *B. thetaiotaomicron* also remained high in the gut of recurrently infected mice.^11,12^ There may be various underlying factors contributing to this phenomenon, including disruptions in the gut microbiota induced by VAN treatment, which subsequently promotes the proliferation of gram-negative bacteria during transition. Additionally, significant alterations occur in the metabolic profiles of amino acids and bile acids within the gut, alongside modifications in the host’s immune response.^11^ It is noteworthy that even at low concentrations, disturbances caused by VAN persistently affect the gut.^11,12^ Therefore, we speculate that there may be a collaborative relationship between *B. thetaiotaomicron* and *C. difficile* in the VAN environment and that this pattern of ecological mutualism may diminish the actual killing effect of the VAN on *C. difficile* or provide support for the survival, colonization, and reinfection of *C. difficile* in the antibiotic environment. In this study, we investigated the ecological interactions and symbiotic biofilm formation between *C. difficile* and *B. thetaiotaomicron* in the VAN environment, and assessed the changes of several important biological indicators of *C. difficile* in the symbiotic biofilm, to reveal the possible associations between some of the recurrent infections of CDI under VAN treatment and these two microbes and their symbiotic biofilms. Meanwhile, the present study also provided some references for the targeted modulation of gut *B. thetaiotaomicron* content to improve the actual efficacy of vancomycin in CDI.

## 2. Materials and methods

### 2.1. Strains and cell culture

*Clostridioides difficile* ATCC 43,255 (VPI 10,463, CD), *Vibrio campbellii* ATCC^Ⓡ^BAA-1117 (VC) was purchased from the American Type Culture Collection (ATCC). *Bacteroides thetaiotaomicron* Z01 (BT) was deposited in our laboratory. CD and BT were cultured in the brain heart infusion (BHI) broth and brain heart infusion-supplemented (BHIS, with 5 mg/L heme chloride) broth (Hopebio, Qingdao, China) at 37°C in an anaerobic workstation (ELACTROTEC, 10% H_2_, 10% CO_2_, 80% N_2_). The VC were activated in the 2216E broth (Hopebio, Qingdao, China) and diluted with freshly prepared Autoinducer Bioassay (AB) broth (17.5 g/L NaCl, 12.3 g/L MgSO_4_, 2.0 g/L acid-hydrolyzed casein, pH 7.5) to the concentration required for the experiments, followed by incubation at 30°C. Vancomycin (VAN) was purchased from Macklin Biochemical Technology Co., Ltd (Macklin, Shanghai, China).

Caco-2 cell line was deposited in our laboratory and was inoculated in Dulbecco’s Modified Eagle’s Medium (DMEM) broth (KeyGEN, Nanjing, China) supplemented with 20% (v/v) fetal bovine serum (FBS, Meisen CTCC, Zhejiang, China), 100 IU/mL penicillin, 100 μg/mL streptomycin, placed in a 37°C incubator (containing 5% CO_2_), cultured overnight and replacement with the culture broth, and when the cells were fused with wall-adherent growth to 80%, digested and passaged with 0.25% (w/v) trypsin solution (KeyGEN, Nanjing, China).

### 2.2. Minimum inhibitory concentration (MIC) of VAN antagonizing CD and BT

The VAN solution (512 μg/mL) was prepared using the twofold dilution method with fresh BHI broth as the solvent.^13^ In 96-well plates the VAN solution was diluted with a gradient of fresh BHI/BHIS broth and 20 μL of CD (or BT) bacterial suspension (OD_600 nm_ close to 0.02) was added to each well. Absorbance was measured by a microplate reader (H1M, BioTek, USA) after these 96-well plates were anaerobic and incubated at 37°C for 48 h. MIC was defined as the lowest VAN concentration that would have the ability to inhibit microbial growth and was significantly different compared to the control (fresh BHI/BHIS broth without VAN).

### 2.3. Determination of growth and biofilm production of CD and BT in the VAN environment

The 1×MIC concentration of VAN antagonizing CD had no apparent effect on BT growth. Thus, the 1×MIC concentration of VAN antagonizing CD was used as a base for gradient dilutions in 96-well plates with fresh BHI broth to obtain different concentrations of VAN solutions (Fig. S1). Mono-cultured: The VAN solution was mixed with 20 μL of CD or BT bacterial solution (OD_600 nm_ close to 0.02) and the final concentration of VAN in the mixed solution was adjusted to MIC-VAN, MIC/4-VAN, and MIC/16-VAN, respectively, with a final volume of 200 μL under all treatments (fresh BHIS broth as solvent). Co-cultured: the VAN solution was mixed with 20 μL of CD bacterial solution (OD_600 nm_ close to 0.02) and 20 μL of BT bacterial solution (OD_600 nm_ close to 0.02), and the final concentration of VAN in the mixed solution was adjusted to MIC-VAN, MIC/4-VAN, and MIC/16-VAN, respectively, with a final volume of 200 μL under all treatments (fresh BHIS broth as solvent). After anaerobic incubation at 37°C for 48 h, the growth of CD and BT was determined at MIC, MIC/4, and MIC/16 concentrations of VAN, respectively. Without the addition of antibiotics, an equal volume of fresh BHIS was used as a control instead. The biofilm was cultured using the same preparation method, and the bacterial cells were discarded after 72 h of anaerobic incubation at 37°C, after which the biofilm content was determined by crystal violet staining and elution according to the method of Yang et al.^14^

### 2.4. Determination of AI-2 content in VAN environment

The culture system was configured regarding the method in the growth and biofilm production assay, and the components were expanded in equal proportions so that the final volume of the culture system was 1 mL. All these treatments were anaerobically incubated at 37°C for 48 h. After that, the bacterial suspension was centrifuged (8000 rpm/min, 4°C, 10 min) and filtered through a sterile filter (0.22 μm), and the supernatant was collected. Bacteria cultured normally without the addition of VAN were used as a control. The AI-2 content in the supernatant was determined by referring to the method of Rui et al.^15^ with slight modifications. The overnight cultured VC bacterial solution was diluted 500-fold with fresh AB broth. After that, the sample supernatant, negative control, medium control supernatant, and the diluted VC bacterial solution were mixed at a volume ratio of 1:10, added to 96-well plates, and incubated at 30°C with 180 r/min oscillation. The fluorescence intensity of each sample was measured at the lowest fluorescence intensity of the negative control and the relative fluorescence intensity was calculated to represent the signaling molecule AI-2 activity. Calculation formula: Relative fluorescence intensity (RLU) = LU_sample_/LU_medium_.

### 2.5. Effect of CD or BT bacterial cell-free supernatant (CFS) in VAN environment on their respective growths

The CD and BT bacterial solution, which were cultured to the stable stage, were centrifuged (12000 rpm/min, 4°C, 10 min) and filtered through a sterile filter (0.22 μm), and the cell-free supernatant (CFS) was collected. Twenty microliters of CD bacterial solution and 180 μL of different concentrations of VAN solution (solvent BT-CFS) were mixed. Twenty microliters of BT bacterial solution and 180 μL of different concentrations of VAN solution (solvent CD-CFS) were mixed. The two treatments were separately added and incubated in 96-well plates, and the final concentration of VAN in each well solution was adjusted to MIC, MIC/4, and MIC/16, respectively. After anaerobic incubation at 37°C for 48 h, OD_600 nm_ was measured by a microplate reader. The control group was cultured with fresh BHI/BHIS without VAN.

### 2.6. Evaluation of bacterial cell swimming motility and c-di-gmp content in the VAN environment

The swimming motility of CD or BT was tested according to the method of Ahmed et al.^16^ Configure the culture system regarding the methods used in the effects of CFS on bacterial growth. All treatments were placed in anaerobic incubation at 37°C for 12 h and then punctured and inoculated on 1/2BHI semi-solid plates (0.5% agar), which were anaerobically incubated at 37°C for 4 d. Colony diameter was measured. The control group was cultured with fresh BHI/BHIS broth without VAN. With the same treatment, one milliliter of the bacterial solution corresponding to the different treatment groups was centrifuged to take its supernatant, and the content of cyclic di-GMP (c-di-GMP) in the supernatant was determined by using a Bacterial c-di-GMP ELISA kit (LMAI Bio, Shanghai, China).

### 2.7. Determination of TcdA and TcdB content in co-culture in the VAN environment

The culture system was configured regarding the methods used in the growth and biofilm yield assays, and the components were expanded in equal proportions to bring the final volume of the culture system to 1 mL. The different treatments were incubated anaerobically at 37°C for 48 h and then centrifuged and filtered to collect the supernatant. Without the addition of antibiotics, an equal volume of fresh BHIS was used as a control instead. TcdA and TcdB content in the supernatant was determined using *C. difficile* toxin A (CDT-A) and toxin B (CDT-B) ELISA kits (Albion, Shanghai, China).

### 2.8. Evaluation of C. difficile virulence gene expression levels in co-cultured by qPCR

Four treatment groups were set up. Mono CD without VAN, which CD was cultured in BHI broth without VAN. Mono CD with VAN, which CD was cultured in BHI broth with MIC-VAN. Co without VAN, which CD was cultured in BT-CFS without MIC-VAN. Co with VAN, which CD was cultured in BT-CFS with MIC-VAN. The culture system was configured regarding the methods used in the growth and biofilm production assays, and the components were expanded in equal proportions so that the final volume of the culture system was 1 mL, and then centrifuged to collect the bacterial cells after anaerobic incubation at 37°C for 48 h. The RNA of CD cells was extracted using the TransZol Up Plus RNA Kit (TransGen Biotech, Beijing, China), followed by DNA contamination removal and reverse transcription of RNA into cDNA using the TransScript One-step gDNA Removal and cDNA Synthesis Supermix kit (TransGen Biotech, Beijing, China), and qPCR was performed to analyze the gene expression levels of *tcdA*, *tcdB*, and *spo0A*. The sequences of primers used for qPCR are shown in Table 1. A two-step amplification method was used with the following PCR reaction conditions: pre-denaturation at 94°C for 30 s, followed by 40 cycles (this process includes denaturation at 94°C for 5 s and annealing at 60°C for 30 s). A lysis curve was added at the end of the qPCR reaction to test for the homogeneity of the amplified fragments. According to the method of Yang et al.,^13^ the 16S rRNA gene was used as the internal reference gene, and the relative quantification of the target gene was performed according to the 2^−ΔΔCt^ method to derive the differential expression folds of the target gene.

**Table 1.** Primers for qPCR.

Primer	Sequence (5’ to 3’)
16S rRNA-F	AGAGTTTGATCCTGGCTCAG
16S rRNA-R	GGTTACCTTGTTACGACTT
tcdA-F	CAGTCGGATTGCAAGTAATTGACAAT
tcdA-R	AGTAGTATCTACTACCATTAACAGTCTGC
tcdB-F	TACAAACAGGTGTATTTAGTACAGAAGATGGA
tcdB-R	CACCTATTTGATTTAGMCCTTTAAAAGC
Spo0A-F	AGCGCAATAAATCTAGGAGCAGA
Spo0A-R	TGGTCTAGGTTTTGGCTCAACT

### 2.9. Evaluation of the killing effect of co-culture on caco-2 cells

The CD group (mono CD), the BT group (mono BT), the CB group (co), and the CB with VAN group (co with VAN) were respectively set up. The culture system was configured according to the method in the growth and biofilm production assay, and the components were expanded isometrically to make the total volume of the culture system 1 mL, and the CFSs of different groups were collected by centrifuging the bacterial solution after anaerobic incubation at 37°C for 12 h. Caco-2 cells (1 × 10^4^) were added into 96-well plates and cultured in an incubator (37°C, 5% CO_2_) for 4 d to make the cells adherent to the wall, 100 μL of CFS (diluted using DMEM complete broth) was added, and incubated in an incubator (37°C, 5% CO_2_) for 12 h. Subsequently, the cell morphology was photographed. The supernatant was discarded and the cells were washed with PBS two times, then DMEM complete broth containing 10% (v/v) cck-8 solution (GOONIE, Guangzhou, China) was added, and incubated in the incubator (37°C, 5% CO_2_) for 2 h. After that, the absorbance was detected at 450 nm by a microplate reader to calculate the cell viability. Calculation formula: cell survival rate = [(As-Ab)/(Ac-Ab)]×100%; As, the absorbance of experimental wells; Ac, the absorbance of control wells; Ab, the absorbance of blank wells.

### 2.10. Adhesion test of C. difficile to caco-2 cells in VAN environment

As above, set up the same four groups. After 12 h of incubation in all groups, the bacterial solution was collected, and the concentration of the bacterial suspension in each group was adjusted to a similar value (OD_600 nm_ close to 0.2), centrifuged and the supernatant discarded, and the bacterial cells were washed with sterile PBS for three times and then resuspended in DMEM complete broth (without antibiotics). After that, 1 mL of bacterial suspension was added to each well of a 12-well plate containing Caco-2 cells, and after anaerobic incubation at 37°C for 2 h, the supernatant was discarded and washed by PBS for 1 time, and then 200 μL of trypsin solution (2.5%, w/v) was added to each well to digest the cells for 3 min, followed by the termination of the digestion by the addition of 600 μL of DMEM complete broth (without antibiotics), and the cells and bacteria were collected by blowing and coated with a dilution on the BHI plates, and the CD numbers were counted after anaerobic incubation for 3 d at 37°C.

### 2.11. Simulated intestinal fluid tolerance test

The CD group (mono CD), the CB group (co), and the CB with VAN group (co with VAN) were set up to configure the culture system according to the method in the growth and biofilm yield assay, and the volumes of each component were expanded in equal proportions so that the final volume of the system was 1 mL. A sterile circular coverslip (to attach the biofilm) was placed at the bottom of the 24-well plate. After 72 h of incubation at 37°C, biofilms attached to coverslips in each group were transferred to simulated intestinal fluid (SIF) containing 1% (w/v) trypsin and 0.15% (w/w) porcine bile salts and placed in an anaerobic incubation for 12 h at 37°C. Then, BHI solid plates were used to count the number of CD viable bacteria in the biofilm before and after the transfer, respectively, to compare the CD survival rate of each group.

### 2.12. Determination of total protein and total polysaccharide in biofilm

A sterile circular coverslip was placed at the bottom of the 24-well plate and set up for CD group (mono CD), BT group (mono BT), CB group (co), and CB with VAN group (co with VAN). The culture system was configured as in the growth and biofilm production assay, and the components were expanded in equal proportions so that the total volume of the culture system was 1 mL. After anaerobic incubation at 37°C for 72 h, the supernatant was discarded, and the biofilm fraction was washed three times with PBS to wash off the floating state of CD and BT cell clusters that had not formed a biofilm. After drying at room temperature, the coverslips were placed in a centrifuge tube containing 1 mL of PBS and sonicated at 37°C for 15 min to disperse the biofilm matrix, after which they were centrifuged (10,000 rpm/min, 4°C, 30 min), and the supernatant was collected for the determination of the contents of proteins and polysaccharides in the biofilm matrix. Determination of polysaccharide content by sulfuric acid-phenol method.^17^ The total protein content in the symbiotic biofilm was determined using a protein content determination assay kit (Jiancheng Bioengineering Institute, Nanjing, China).

### 2.13. Scanning electron microscopy (SEM) combined with confocal laser scanning microscopy (CLSM) to analyze symbiotic biofilms under co-culture

A sterile circular coverslip was placed at the bottom of the 24-well plate and set up for the CD group (mono CD), BT group (mono BT), CB group (co), and CB with VAN group (co with VAN). The culture system was configured as in the growth and biofilm production assay, and the components were expanded in equal proportions so that the total volume of the culture system was 1 mL. After anaerobic incubation at 37°C for 72 h, the cells were washed once with PBS, and the bacterial cells were inserted into 24-well plates containing 2 mL of glutaraldehyde solution (2.5%) to fix the morphology of the bacterial cells, and the cells were incubated at 4°C overnight, and then a gradient of dehydration was carried out by adding 25%, 50%, 75%, 95%, and 100% ethanol, and the dehydration lasted for 15 min each time, and the dehydration was repeated twice. Afterward, the samples were freeze-dried (−60°C, 0 Pa) in a vacuum freeze-dryer (Sihuan Qihang Technology, Beijing, China). The samples were then sprayed with gold and observed in a scanning electron microscope (Apreo 2S, Thermo Scientific, USA) and images were captured using Inletex Easy Meeting Classic Pro software.

Biofilms were cultured according to the same method. After anaerobic incubation at 37°C for 72 h, the formed biofilm was washed with PBS, and a 2000-fold dilution of SYTO Green (KGI Bio, Nanjing, China) was added, incubated in the dark room for 1 h and then washed with PBS, and the samples with the biofilm side facing down were covered on 20 mm × 20 mm coverslips and placed under a confocal laser scanning microscopy (CLSM) (AX Confocal Microscope System, Nikon, Japan) equipped with a × 60 oil-immersion lens, and scanned the biofilm in the Z-axis direction (Ex/Em = 500/530 nm).

### 2.14. Fluorescence in situ hybridization (FISH) probes to assess CD and BT distribution in symbiotic biofilms

With slight modifications to the method of Xiao et al.^18^ A sterile circular coverslip was placed at the bottom of the 24-well plate and set up the CB group (co), and the CB with VAN group (co with VAN), respectively. The culture system was configured as in the growth and biofilm production assay, and the components were expanded in equal proportions so that the total volume of the culture system was 1 mL. After anaerobic incubation at 37°C for 72 h, the formed biofilm was washed with PBS and fixed at 4°C for 12 h by adding 4% paraformaldehyde solution. The samples were washed with PBS and then PBS containing 50% ethanol was added and fixed for 12 h at 4°C. 100 ng of each of the two specific probes (Sangong Bioengineering, Shanghai, China) was added to 20 μL of hybridization solution (6×SSC; 5×Denhardt; 0.5% SDS; 100 μg/ml salmon sperm DNA; 50% formamide), dropped at the biofilm of the slides, and incubated at 46°C for 2 h. The elution solution (20 mmol/L Tris/HCl, 5 mmol/L EDTA, 159 mmol/L NaCl, 0.01% (w/v) SDS, pH 7.5) was used to repeatedly elute the excess probe, and incubated at 48°C for 15 min. The FISH probes (Bioengineering, Shanghai, China) used in this study were as follows: CD-Specific probes, 5′- CATCCTGTACTGGCTCAC-3′, 5′ modified with Cy3, Ex/Em = 550/570 nm; BT-Specific probes, 5′- CATTTGCCTTGCGGCTA-3′, 5′ modified with Cy5, Ex/Em = 640/670 nm.^19,20^

### 2.15. Statistic analysis

All experiments were performed using three independent biological replicates. Statistical analysis and graphing of data using GraphPad Prism 8.4.3. Growth, biofilm production, AI-2 content, bacterial mobility diameter, cell viability, cell adhesion, simulated intestinal fluid tolerance, biofilm thickness, biofilm matrix polysaccharide, protein content, and the number of CD cells in the biofilm were analyzed using Tukey’s multiple comparisons tests. TcdA and TcdB toxin production, c-di-GMP content, and virulence gene expression levels were analyzed using two-way ANOVA followed by Sidak’s multiple comparisons test, and results at *p* < 0.05 were considered significantly different.

## 3. Results

### 3.1. CD and BT symbiotic biofilm production is higher than CD monophyletic biofilm production in the VAN environment

**Figure 1.**
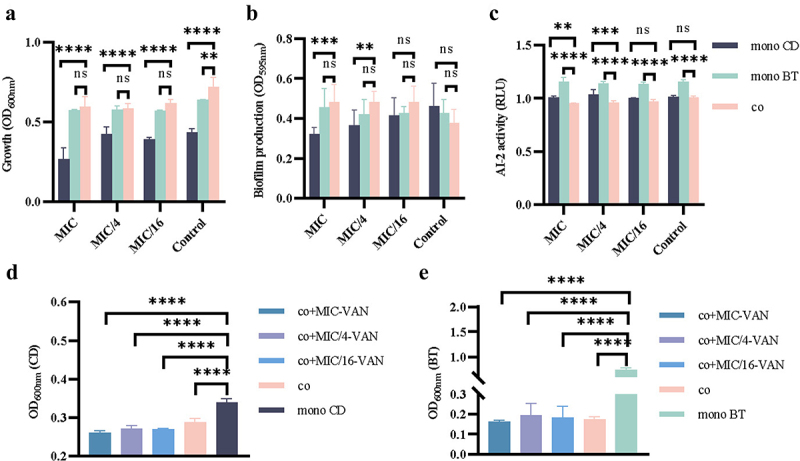
Growth, biofilm production, and AI-2 production of CD and BT alone and in co-culture under VAN environment. (a) Growth (biomass) of CD and BT when grown mono- and co-cultured in different concentrations of VAN environment. co, co-cultured of CD and BT. Control, mono-culture, or co-culture without VAN. (b) Biofilm production of CD and BT when grown mono- and co-cultured in different concentrations of VAN environment. co, co-cultured of CD and BT. (c) AI-2 content in fermentation supernatants of CD and BT mono- and co-cultured in different concentrations of VAN environments. co, co-cultured of CD and BT. (d) Effect of BT cell-free supernatant (BT-CFS) on CD growth in different concentrations of VAN environment. co, co-cultured of CD and BT-CFS. co+MIC-VAN, co-culture of CD and BT-CFS in MIC-VAN environment. co+MIC/4-VAN, co-culture of CD and BT-CFS in MIC/4-VAN environment. co+MIC/16-VAN, co-culture of CD with BT-CFS in MIC/16-VAN environment. E. Effect of CD cell-free supernatant (CD-CFS) on BT growth in different concentrations of VAN environment. co, co-cultured of BT and CD-CFS. co+MIC-VAN, co-culture of BT and CD-CFS in MIC-VAN environment. co+MIC/4-VAN, co-culture of BT and CD-CFS in MIC/4-VAN environment. co+MIC/16-VAN, co-culture of BT and CD-CFS in MIC/16-VAN environment. mono CD, CD mono-cultured. mono BT, BT mono-cultured. The MIC value was 0.5μg/mL. Significant differences were analyzed using two-way ANOVA followed by Sidak’s multiple comparisons test. ns, *p* > 0.05. **, *p* < 0.01. ***, *p* < 0.001. ****, *p* < 0.0001.

The MIC values of VAN antagonizing CD and BT were 0.5 μg/mL and 1 μg/mL, respectively. The 1×MIC concentration of VAN antagonizing CD had no apparent effect on BT growth. Thus, the 1×MIC concentration of VAN antagonizing CD (0.5 μg/mL) was used as a base for gradient dilutions in 96-well plates with fresh BHI broth to obtain different concentrations of VAN solutions. Using a gradient dilution based on the concentration of 1×MIC-VAN (0.5 μg/mL) antagonizing CD, we found that the biomass (growth) produced by co-culture of CD and BT was higher than that of CD alone at different concentrations of VAN (Figure 1(a)), and the symbiotic biofilm production was also higher than that of CD alone in the VAN environment, especially at the MIC and the 1/4MIC-VAN (Figure 1(b)). However, the biomass of CD and BT co-cultured in the absence of VAN was higher than that of the monoculture, but their symbiotic biofilm production was lower than that of the two alone. Regardless of the presence or absence of VAN, AI-2 content was lower under the co-culture system than in monoculture, and the difference was particularly significant at MIC versus 1/4MIC-VAN (Figure 1(c)). We further evaluated the effect of cell-free supernatant (CFS) of the two microbes on their respective growth and found that in the absence of VAN, the CFS of CD and BT showed mutual inhibition of each other’s growth; whereas the biomass of CD was further reduced in the co-culture in the presence of VAN, but not that of BT in the co-culture (Figures 1(d) and 1(e)). Taken together, the symbiotic biofilm production formed by the co-culture of CD and BT in the VAN environment was higher.

### 3.2. Enhanced bacterial cell swimming ability, elevated toxin and c-di-gmp content, and up-regulated expression levels of virulence genes tcdA and tcdB in co-culture under VAN environment

**Figure 2.**
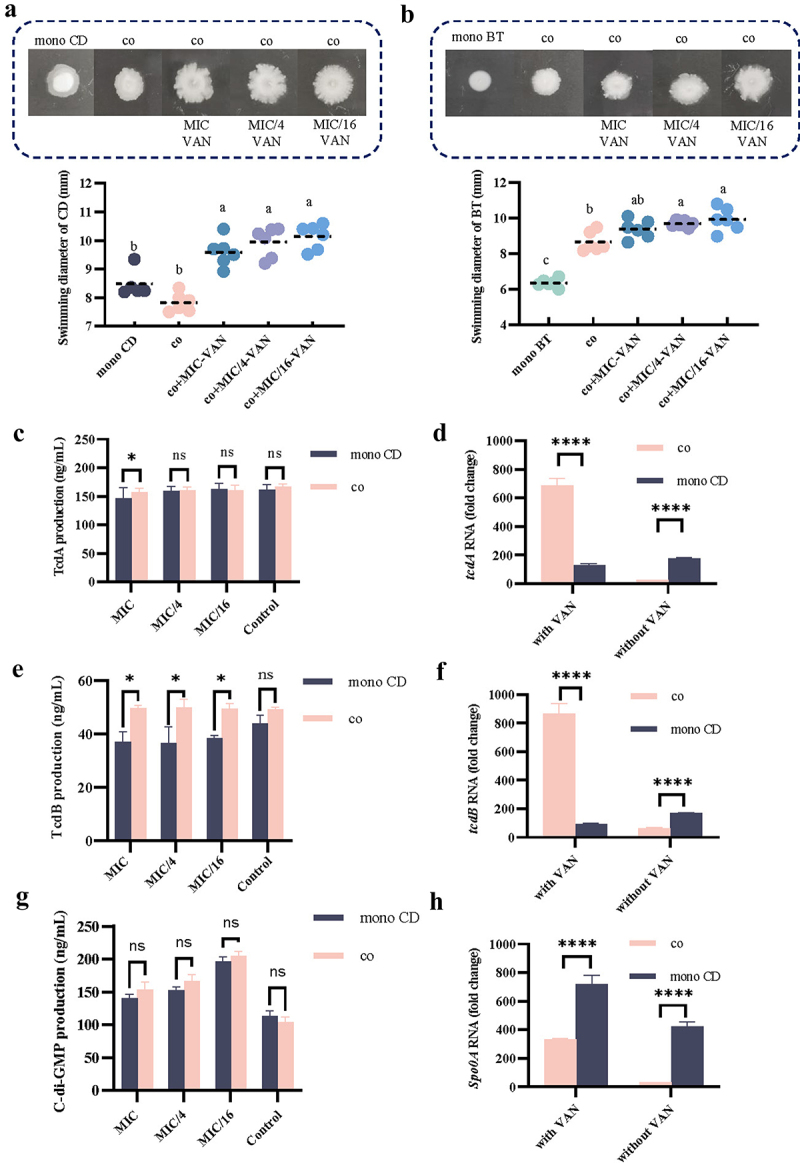
Bacterial cell swimming motility, toxin protein and c-di-gmp production, and relative expression levels of virulence genes *tcdA*, *tcdB*, and *spo0A*. (a) Bacterial mobility of CD mono-culture and the co-cultured of CD and BT-CFS in VAN environment. co, co-cultured of CD and BT-CFS. co+MIC-VAN, co-culture of CD and BT-CFS in MIC-VAN environment. co+MIC/4-VAN, co-culture of CD and BT-CFS in MIC/4-VAN environment. co+MIC/16-VAN, co-culture of CD and BT-CFS in MIC/16-VAN environment. (b) Bacterial mobility of BT mono-culture and co-cultured BT and CD-CFS in VAN environment. co, co-cultured of BT and CD-CFS. co+MIC-VAN, co-culture of BT and CD-CFS in MIC-VAN environment. co+MIC/4-VAN, co-culture of BT and CD-CFS in MIC/4-VAN environment. co+MIC/16-VAN, co-culture of BT and CD-CFS in MIC/16-VAN treatment. (c) TcdA production in CD mono-culture and co-cultured of CD and BT in VAN environment. co, co-culture of CD and BT. Control, CD mono-culture without VAN or CD, and co-culture of CD and BT without VAN. (d) Changes in the relative expression level of the *tcdA*. co, co-cultured of CD and BT-CFS. (e) TcdB production in CD mono-culture and co-cultured of CD and BT in VAN environment. co, co-culture of CD and BT. Control, CD mono-culture without VAN or CD, and co-culture of CD and BT without VAN. (f) Changes in the relative expression level of the *tcdB*. co, co-cultured of CD and BT-CFS. (g) c-di-GMP production in CD mono-culture and co-cultured of CD and BT in VAN environment. co, co-culture of CD and BT. Control, CD mono-culture without VAN or CD, and co-culture of CD and BT without VAN. (h) Changes in the relative expression level of the *Spo0A*. co, co-cultured of CD and BT-CFS. mono CD, CD mono-cultured; mono BT, BT mono-cultured. The MIC value was 0.5μg/mL. Significant differences were analyzed in Figures 2(a) and 2(b) using Tukey’s multiple comparisons test, where different lowercase letters represent significant differences, *p* < 0.05. Significant differences were analyzed in Figures 2(c), 2(d), 2(e), 2(f), 2(g), and 2(h) using two-way ANOVA followed by Sidak’s multiple comparisons test. ns, *p* > 0.05. *, *p* < 0.05. ****, *p* < 0.0001.

In the co-culture system without VAN, CD cell swimming motility was reduced, however, with the addition of VAN, this ability was increased (Figure 2(a)). For BT, its swimming motility was enhanced in co-culture with or without VAN (Figure 2(b)). We further determined the changes in the content of the toxin protein TcdA and TcdB produced by CD in co-culture with or without VAN, and found that the addition of MIC-VAN inhibited TcdA and TcdB production when CD was mono-cultured, whereas the production of TcdA and TcdB in the co-cultured was higher than that in the mono-cultured, especially the TcdB level (Figures 2(c), 2(e)). The relative expression levels of *tcdA*, *tcdB*, and *spo0A* in CD cells in co-culture without VAN were reduced compared to CD in mono-culture, while the relative expression level of *tcdA* was up-regulated 5.5-fold and the relative expression of *tcdB* gene was up-regulated 9.4-fold in CD cells that were co-cultured in VAN environment compared to CD mono-culture (Figures 2(d), 2(f), 2(h)). Simultaneously, we found that c-di-GMP content was not dramatically different in mono-culture and co-culture, but was remarkably higher in the presence of MIC-VAN, especially in the 1/16 MIC-VAN environment (Figure 2(g)). Changes in c-di-GMP content appeared to be positively correlated with bacterial cell swimming ability. These results indicated that in the co-culture system in the presence of VAN, the swimming motility of the CD cells, the toxin protein production, and the c-di-GMP content were enhanced, and the expression levels of *tcdA* and *tcdB* were up-regulated.

### 3.3. The killing effect of co-culture system on caco-2 in VAN environment and evaluation of the adhesion of CD to caco-2 cells

**Figure 3.**
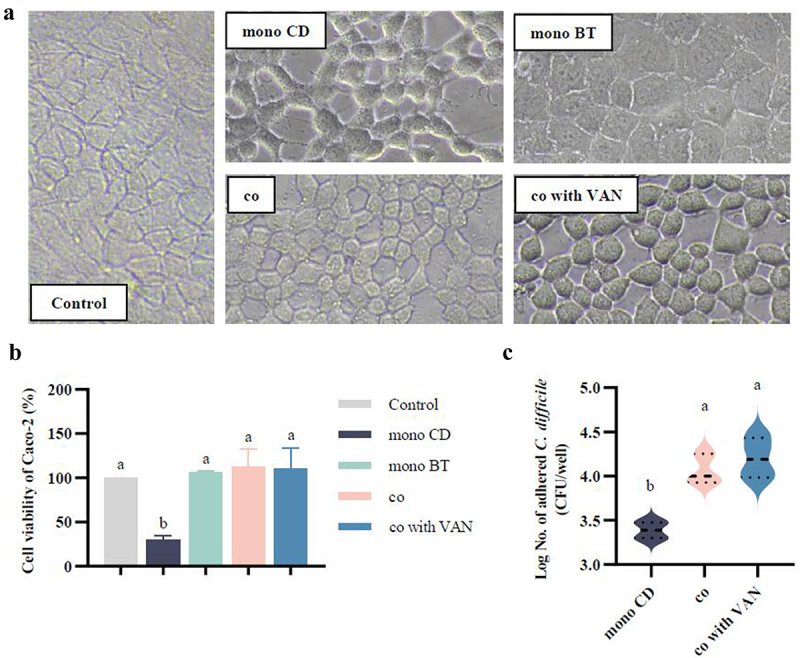
Effect of the co-culture system on caco-2 cell activity and assessment of the effect of CD adhesion to caco-2 in VAN environment. (a) Effect of different treatments on the morphology of Caco-2 cells. Control, the effect of 4-fold dilution of BHIS on Caco-2 cell morphology. mono CD, the effect of CD mono-cultured on Caco-2 cell morphology. mono BT, the effect of BT mono-cultured on Caco-2 cell morphology. co, the effect of co-cultured CD and BT on Caco-2 cell morphology. co with VAN, the effect of co-culture of CD and BT on Caco-2 cell morphology in the VAN environment. (b) Caco-2 cell survival under different treatments. Control, Caco-2 cell survival under BHIS treatment at 4-fold dilution. mono CD, the effect of CD mono-culture on Caco-2 cell survival. mono BT, the effect of BT mono-culture on Caco-2 cell survival. co, the effect of co-cultured CD and BT on Caco-2 cell survival. co with VAN, the effect of co-culture of CD and BT on Caco-2 cell survival in the VAN environment. (c) Evaluation of CD adhesion rate to Caco-2 cells under different treatments. mono CD, the effect of CD mono-culture on Caco-2 cell adhesion rate; co, effect of co-cultured of CD and BT on Caco-2 cell adhesion rate; co with VAN, the effect of co-cultured of CD and BT on Caco-2 cell adhesion rate in VAN environment. Significant differences were analyzed using Tukey’s multiple comparisons test, with different lowercase letters representing significant differences at *p* < 0.05.

Compared to the control, i.e., Caco-2 cells treated with a four-fold dilution of BHI for 2 h, CD in mono-culture significantly reduced Caco-2 cell viability (29.6%), induced cell shrinkage and rounding, and blurred cell edges. In contrast, BT did not exhibit a significant killing effect on Caco-2 cells, and the cells showed a tightly junctional state under its treatment, similar to the morphology of control cells (Figures 3(a) and 3(b)). In both co-culture with and without VAN environment, Caco-2 cells survival reached 100%, but there were slight differences in cell morphology (Figure 3(a)). Compared with the co-culture without VAN, the morphology of Caco-2 cells in the co-culture with VAN was rounded and contracted, and the cell gap was slightly widened. Meanwhile, the adhesion data showed that the CD adhesion capacity to Caco-2 cells under the co-culture (1.18 × 10^4^ CFU/well) was much higher than that of CD mono-culture to Caco-2 cells (2.45 × 10^3^ CFU/well). This adhesion was further improved with the addition of VAN in co-culture, and the adhesion capacity of CD to Caco-2 cells reached 1.6 × 10^4^ CFU/well (Figure 3(c)). The above results indicated that the killing effect of CD for Caco-2 cells under the co-culture system containing VAN, although decreased, prompted a substantial increase in its ability to adhere to cells.

### 3.4. Survival of CD cells in symbiotic biofilms in simulated intestinal fluids and determination of protein and polysaccharide content in symbiotic biofilm

**Figure 4.**
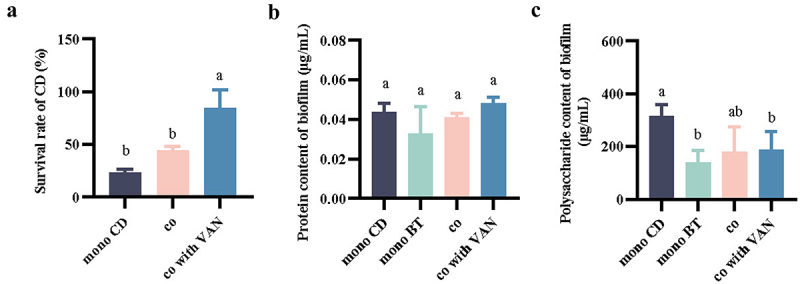
Survival of CD in symbiotic biofilm in simulated intestinal fluid and determination of symbiotic biofilm protein and polysaccharide content. (a) Survival of CD in monophyletic or symbiotic biofilms in simulated intestinal fluid. (b) Protein content in monophyletic or symbiotic biofilms. (c) Polysaccharide content in monophyletic or symbiotic biofilm matrices. Note: mono CD, CD monophyletic biofilm; mono BT, BT monophyletic biofilm; co, symbiotic biofilm formed by CD and BT; co with VAN, symbiotic biofilm formed by CD and BT in VAN environment. Significant differences were analyzed using Tukey’s multiple comparisons test, with different lowercase letters representing significant differences at *p* < 0.05.

Comprehensive growth, biofilm production, Caco-2 cell adhesion, and many other data, it was found that the survival rate of CD under the symbiotic system was significantly improved, which may be closely related to the formation of symbiotic biofilm. We used artificial simulated intestinal fluid to treat the symbiotic biofilms formed under co-culture and found that the CD cell survival rate in the symbiotic biofilms was elevated compared to that in the mono-culture biofilms (from 27.8% to 44.7%), and the CD survival rate in the symbiotic biofilms was further increased with the addition of VAN (reached 86.3%) (Figure 4(a)). To observe the changes in biofilm matrix content, we determined the major components, i.e., polysaccharide and protein content, in the matrix of monophyletic and symbiotic biofilms formed in VAN-free and VAN-containing environments and the matrix of symbiotic biofilms formed in VAN-containing environments. In terms of protein content, there was no significant difference between monophyletic and symbiotic biofilm matrices (with or without VAN) (Figure 4(b)). In terms of polysaccharide content, the polysaccharide content in the CD monoculture biofilm matrix (316.65 ± 42.29 μg/mL) was slightly higher than that in the symbiotic biofilm matrix (179.99 ± 96.27 μg/mL) (Figure 4(c)). The polysaccharide content in the symbiotic biofilm matrix formed in the VAN environment was slightly higher than that in the symbiotic biofilm matrix formed in the VAN-free environment (Figure 4(c)). Overall, the protein and polysaccharide contents of the symbiotic biofilm matrix formed by CD and BT in the VAN environment did not show significant differences compared to the other treatment groups, but the number of CD viable bacteria in it was significantly higher compared to the number of CD viable bacteria in the monoculture biofilm.

### 3.5. Morphological changes of symbiotic biofilms in co-culture system in VAN environment observed by SEM and CLSM

**Figure 5.**
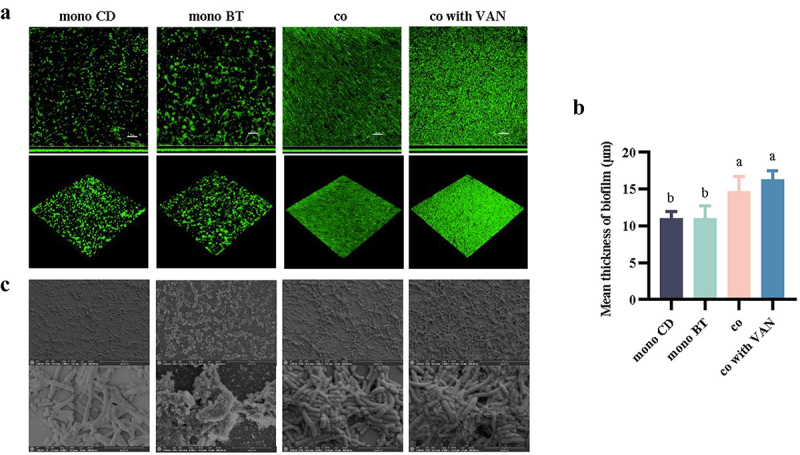
Imaging of biofilms of CD or BT mono-cultured and when co-cultured in VAN environment under laser confocal (CLSM) and scanning electron microscopy (SEM). (a) Laser confocal analysis of 3D images of monophyletic and symbiotic biofilms. Scale: 25μm. (b) Thickness of monophytic and symbiotic biofilms (μm). (c) SEM images of monophyletic and symbiotic biofilms, scale bar: 50μm (top), 5μm (bottom). Note: mono CD, CD monophyletic biofilm; mono BT, BT monophyletic biofilm; co, symbiotic biofilm formed by CD and BT; co with VAN, symbiotic biofilm formed by CD and BT in VAN environment. Significant differences were analyzed using Tukey’s multiple comparisons test, with different lowercase letters representing significant differences at *p* < 0.05.

CLSM imaging showed that CD and BT when cultured alone produced irregular, sparse, biofilms with a three-dimensional structure containing macroaggregates and short planktonic cells. In contrast, the mixed culture of CD and BT produced a thick and homogeneous symbiotic biofilm, dense, carpet-like, with many planktonic cells and a low three-dimensional structure. Symbiotic biofilms produced in VAN environments were thick, regular, dense, carpet-like, and consisted of densely arranged contiguous macroaggregates with a high degree of three-dimensional structure, with marked elongation of planktonic cells and macroaggregates (Figure 5(a)). Moreover, the average thickness of CD and BT monophyletic biofilms was 11.00 ± 1.00 μm and 11.00 ± 1.73 μm, respectively, while that of CD and BT symbiotic biofilms was 14.67 ± 2.08 μm, and that of CD and BT symbiotic biofilms in the VAN environment was 16.33 ± 1.15 μm, and symbiotic biofilms in VAN environment were significantly thicker as compared to those of CD and BT monophyletic biofilm was significantly thicker (Figure 5(b)). Bacteria in the biofilm were observed to be encapsulated in the extracellular matrix under SEM, and the bacteria in the symbiotic biofilm formed under VAN environment were tightly arranged and intertwined to form large bacterial clusters. In contrast, the number of bacteria in the symbiotic biofilm formed in the VAN-free environment was reduced, but many bacterial clusters were still tightly intertwined. Monophyletic biofilms contained fewer bacteria, with increased gaps between the bacteria and reduced agglomeration (Figure 5(c)). Taken together, the thickness of the symbiotic biofilm formed in the VAN environment was elevated, and the number of bacteria was increased.

### 3.6. Fluorescence in situ hybridization (FISH) probes to assess the distribution of CD and BT in symbiotic biofilms

**Figure 6.**
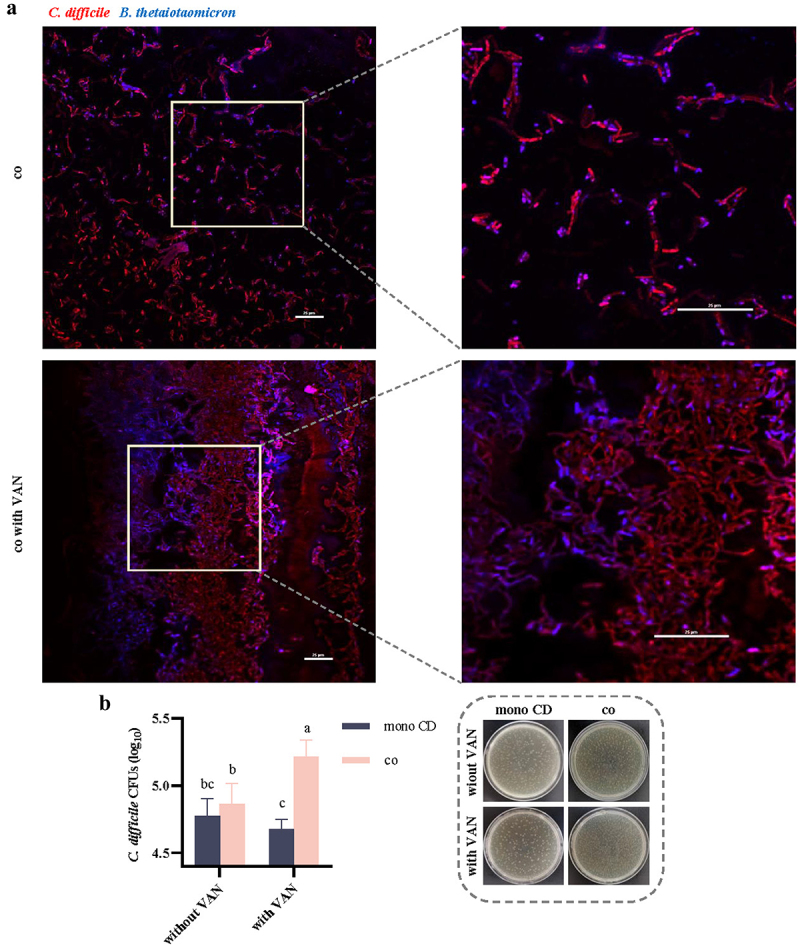
Distribution of CD and BT in symbiotic biofilms labeled by FISH probes and counts of CD viable bacteria in symbiotic biofilms. (a) Distribution of CD and BT in symbiotic biofilms labeled by FISH probes, red fluorescence for Cy3-labeled CD and blue fluorescence for Cy5-labeled BT, scale bar: 25μm. (b) Number of CD cells in CD monophytic and symbiotic biofilms. mono CD, CD monophyletic biofilm; co, symbiotic biofilm formed by CD and BT; co with VAN, symbiotic biofilm formed by CD and BT in VAN environment. Significant differences were analyzed using Tukey’s multiple comparisons test, with different lowercase letters representing significant differences at *p* < 0.05.

To observe the distribution of CD and BT in the symbiotic biofilms formed in VAN-containing and VAN-free environments, we labeled CD and BT in the symbiotic biofilms with FISH probes with different fluorescence. The results showed that CD and BT formed a homogeneous symbiotic biofilm and both microbes were evenly distributed in the symbiotic biofilm. In the symbiotic biofilms formed in the VAN environment, CD and BT formed larger clusters of mixed bacterial cells, and the amount of CD in the symbiotic biofilms was significantly higher (Figure 6(a)). At the same time, we counted the colonies of CD in CD monophyletic biofilms and symbiotic biofilms. In the VAN-free environment, the CD colony counts in CD mono-culture biofilms and symbiotic biofilms were 6.13 × 10^4^ ± 1.79 × 10^4^ CFU/mL and 7.61 × 10^4^ ± 2.76 × 10^4^ CFU/mL, respectively; whereas in the VAN environment, the CD colony counts in CD monoculture biofilms and symbiotic biofilms were 4.79 × 10^4^ ± 0.86 × 10^4^ CFU/mL and 1.68 × 10^5^ ± 0.44 × 10^5^ CFU/mL, respectively (Figure 6(b)). Overall, the colony counts were consistent with the FISH probe results that the symbiotic biofilms formed in the VAN environment contained a greater number of CD cells.

## 4. Discussion

VAN and fidaxomicin (FID) are the antibiotics of choice for the treatment of CDI or rCDI, especially the former, but it is accompanied by a high rate of recurrence, and the main reasons behind this phenomenon include the formation of stubborn spores, the emergence of resistant strains, and opportunistic microbial infections due to disturbances in gut ecology.^21^ Against the backdrop of a slowing pace of discovery of novel antibiotics, traditional antibiotics are still needed to help the human body defend itself against a wide range of microbial infections, which means that it is particularly important to “secondary development” traditional antibiotics. Studies have shown that changes in the structure of gut microbiota can significantly affect the actual efficacy of antibiotics.^22^ Artificial modulation of the structure of the microbiota is feasible in the treatment of a variety of diseases, such as the use of fecal microbiota transplantation (FMT) in inflammatory bowel disease (IBD), irritable bowel syndrome (IBS), and CDI.^23,24^ The core of this micro-ecological therapy lies in the precise regulation of the ecological structure of the microbes in the host’s gut to achieve the desired therapeutic effect. The primary site of action of VAN for CDI is in the gut, and we speculate that the presence of specific microbes in the gut may affect the actual efficacy of VAN, ultimately resulting in either very good or poor efficacy. Therefore, improving the efficacy of VAN by artificially adding or deleting specific microbes in the gut may be a new direction for “secondary development” research on traditional antibiotics. However, the development of such therapeutic approaches that modulate the structure of the microbial community and thereby achieve efficacy is predicated on elucidating the ecological relationships between specific members of the gut, and in particular the changes in the biological behavior of microbes in the environment where specific antibiotics are present.

In a previous study, we found that some of the pCDI mice that received VAN treatment reemerged with infections after some time and were accompanied by a significant increase in the relative abundance of *Bacteroides* phylum in the gut, and in particular, of BT among them.^12^ We speculate that there may be an interplay between CD and BT in the VAN environment. Elahi et al. found that BT cell wall-associated glycans inhibit the production of CD glycosylated toxins, thereby curbing the development of CDI.^25^ Similarly, Li et al. found that BT alleviated CDI-induced injury and inflammation by altering the microbial structure and bile acid levels in mice.^26^ Therefore, BT is currently recognized as a next-generation probiotic strain with potential application in CDI control.^27^ However, our findings challenge the notion that the antagonistic relationship between CD and BT may be transformed into a mutually beneficial symbiotic relationship under conditions where VAN is present. We found that in the presence of a specific concentration of VAN, the production of symbiotic biofilms formed by the symbiosis of CD and BT was substantially increased compared to the monophyletic biofilms formed by the growth of CD alone, and these symbiotic biofilms contained a greater number of CD cells within it. At the same time, CD sheltered by symbiotic biofilms showed greater tolerance to intestinal fluids.

Normington et al. found that biofilms weaken the killing effect of VAN on CD and provide continuous shelter, which provides immediate conditions for recurrent infection.^28^ Some gut commensal microbes can interact with CD and thus increase their biofilm production. These symbiotic microbes can be categorized into three groups based on their role in influencing CD biofilm formation, i.e., antagonistic (symbiotic microbes reduce CD biofilm formation), cooperative (the sum of individual microbial biomass is equal to that of the co-cultivated biofilm), and synergistic (the symbiotic microbes increase CD biofilm production).^28^ This study reveals that the relationship between CD and BT in a VAN environment is synergistic. The majority of *C. difficile* clinical isolates secrete two toxin proteins, TcdA and TcdB, which synergistically disrupt the intestinal epithelial structure and thereby cause intestinal tissue damage.^29^ We found that the production of toxin proteins from CD in the symbiotic biofilms formed under VAN induction was enhanced, in particular, the TcdB content was dramatically elevated. It is noteworthy that the trend of increasing protein content of these two toxins is positively correlated with the expression levels of *tcdA* and *tcdB*. In addition, under the symbiotic biofilm, VAN contributed to the enhanced swimming ability of the bacterium (Figure 2(a)), which was accompanied by elevated c-di-GMP content (Figure 2(g)). Changes in c-di-GMP content are critical for CD cell swimming, virulence gene expression levels, flagellar and toxin protein synthesis, and biofilm formation.^30^ It has been shown that elevated levels of c-di-GMP promote biofilm formation, while low levels of c-di-GMP affect bacterial motility property.^31^ In the present study, it was seen that the c-di-GMP content under the symbiotic biofilm was increased in the VAN-induced environment, and this trend was accompanied by an increase in CD cell swimming, CD cell adhesion to Caco-2 cells, and CD cell tolerance to simulated intestinal fluids. However, this seemingly cooperative-like relationship shifted to an antagonistic one in the absence of VAN. These results also further suggested that the ecological relationship between CD and BT is not unique and may be altered by the direct influence of different environmental conditions in the gut. Low concentrations of antibiotics have been shown to stimulate CD to secrete more toxins and promote the formation of its biofilm, which in turn enhances CD adhesion to intestinal epithelial cells.^21,32^ We found that the symbiotic biofilm formed by CD and BT in the VAN environment was dense and thicker, while the adhesion to Caco-2 was significantly higher. Therefore, it can be inferred that the symbiotic biofilm not only improves the resistance of CD in the gut but also promotes the adhesion of CD cells to intestinal epithelial cells. Although the present study has identified an ecological mutualistic relationship between CD and BT under the influence of VAN, further substantial evidence is required to substantiate this finding. In subsequent investigations, we intend to employ antibodies targeting bacterial flagellin or utilize CRISPR-Cas 9/Cpf1 gene editing for targeted knockdown of key genes to gain mechanistic insights into this ecological mutualism.

Taken together, the production of symbiotic biofilms formed by CD and BT in the VAN environment was significantly higher than that of monophyletic biofilms. These symbiotic biofilms contain a greater number of CD cells and are better able to tolerate intestinal fluid killing. Symbiotic biofilms showed increased production of the toxin protein TcdA and TcdB, elevated expression levels of the virulence genes *tcdA* and *tcdB*, and enhanced bacterial swimming and c-di-GMP content, in addition to a substantial increase in the adhesion of CD to Caco-2 cells. BT has the potential to be a next-generation probiotic, but its safety in CDI treatment needs to be further evaluated. At the same time, targeted deletion or attenuation of BT content in the host gut may be more favorable to the actual efficacy of VAN in CDI therapy.

## Supplementary Material

Supplemental Material
