# Supplementary material for: Symbiotic biofilms formed by Clostridioides difficile and bacteroides thetaiotaomicron in the presence of vancomycin
Source: Gut Microbes. 2024 Aug 12;16(1):2390133. doi: 10.1080/19490976.2024.2390133 (PMC11321409; doi:10.1080/19490976.2024.2390133)
Supplement: Supplemental Material [file KGMI_A_2390133_SM0166.zip › Supplementary Information.docx]

**Supplementary Information**


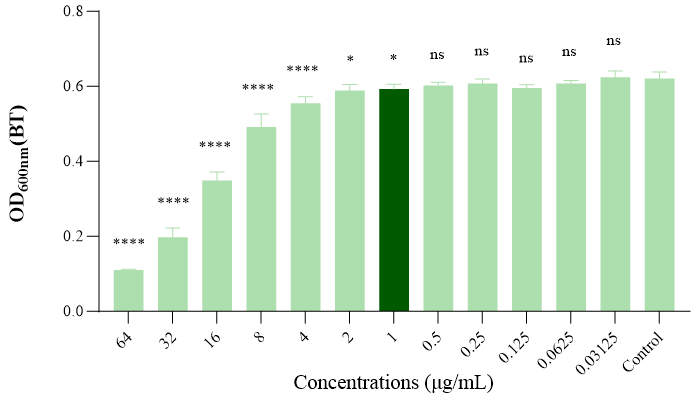

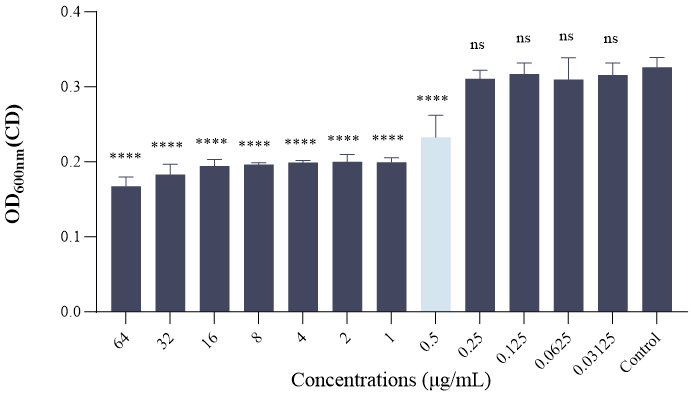


**Fig. S1**. Evaluation of the minimum inhibitory concentration (MIC) of CD and BT antagonized by VAN. Control indicates normal growth. Significant differences were analyzed using two-way ANOVA followed by Sidak's multiple comparisons test. ns, p>0.05. *, p<0.05. **, p<0.01. ***, p<0.001. ****, p<0.0001.
